# Usability Testing of a Patient-Centered Mobile Health App for Supporting and Guiding the Pediatric Emergency Department Patient Journey: Mixed Methods Study

**DOI:** 10.2196/25540

**Published:** 2022-03-15

**Authors:** Jessica Rochat, Frédéric Ehrler, Johan N Siebert, Arnaud Ricci, Victor Garretas Ruiz, Christian Lovis

**Affiliations:** 1 Faculty of Medicine University of Geneva Geneva Switzerland; 2 Division of Medical Information Sciences University Hospitals of Geneva Geneva Switzerland; 3 Department of Pediatric Emergency Medicine Geneva Children's Hospital University Hospitals of Geneva Geneva Switzerland

**Keywords:** usability, user-centered design, information systems, mobile apps, emergency service, hospital, pediatrics, mobile phone

## Abstract

**Background:**

Patient experience in emergency departments (EDs) remains often suboptimal and can be a source of stress, particularly in pediatric settings. In an attempt to support patients and their families before, during, and after their visit to a pediatric ED, a mobile health (mHealth) app was developed by a multidisciplinary team based on patient-centered care principles.

**Objective:**

This study aims to evaluate the usability (effectiveness, efficiency, and satisfaction) of a new mHealth app, *InfoKids*, by potential end users through usability testing.

**Methods:**

The app was assessed through an in-laboratory, video-recorded evaluation in which participants had to execute 9 goal-oriented tasks, ranging from account creation to the reception of a diagnostic sheet at the end of the emergency care episode. Effectiveness was measured based on the task completion rate, efficiency on time on task, and user satisfaction according to answers to the System Usability Scale questionnaire. *Think-aloud* usability sessions were also transcribed and analyzed. Usability problems were rated for their severity and categorized according to ergonomic criteria.

**Results:**

A total of 17 parents participated in the study. The overall completion rate was 97.4% (149/153). Overall, they reported good effectiveness, with the task successfully completed in 88.2% (135/153) of cases (95% CI 83%-93%). Each task, with the exception of the first, created difficulties for some participants but did not prevent their completion by most participants. Users reported an overall good to excellent perceived usability of the app. However, ergonomic evaluation identified 14 usability problems occurring 81 time. Among these, 50% (7/14) were serious as their severity was rated as either *major* or *catastrophic* and indicated areas of improvements for the app. Following the suggested usability improvements by participants, mitigation measures were listed to further improve the app and avoid barriers to its adoption.

**Conclusions:**

Usability of the InfoKids app was evaluated as good to excellent by users. Areas of improvement were identified, and mitigation measures were proposed to inform its development toward a universal app for all ED patients visiting a digitalized institution. Its contribution could also be useful in paving the way for further research on mobile apps aimed at supporting and accompanying patients in their care episodes, as research in this area is scarce.

## Introduction

### Background

An emergency department (ED) visit is often the first point of contact for patients with a health care institution and thus a showcase of its efficiency. Providing patients with a positive experience should take high priority [1] and is one of the fundamental determinants of health care quality [2]. In a recent meta-synthesis, a study by Graham et al [1] conceptualized a model to understand the most commonly identified drivers of the ED patient experience. These included interpersonal and informational communication, patients’ expectations and empowerment, recognition of emotional needs, actual and perceived waiting times, competent care, and physical and environmental needs [1]. A similar conceptual framework was also developed by Sonis et al [3,4]. The same drivers have been observed in other studies focusing on identifying the determinants of patient and family experience in the pediatric EDs [5-11]. This highlights the essential nature of these drivers and the attention that should be paid to them when implementing an intervention to improve the adult or pediatric ED patient experience and ED efficiency. Several recent reviews have demonstrated a strong correlation between a positive ED patient experience and a range of benefits at the individual and institutional levels. These include increased therapeutic compliance [12]; improved health clinical outcomes [1,13,14]; outpatient [15], inpatient [16], and staff satisfaction [12]; reduced complaints and medicolegal risks [17]; institutional profitability and reputation in the community [12,18,19]; and other health care system goals [13].

Unfortunately, the hectic, unpredictable, crowded, demanding, and time-pressured environment of the ED may adversely affect patient experience [13]. In particular, there is strong pressure from public and institutional leaders to alleviate overcrowding and long waiting times experienced in the ED [20]. Overcrowding because of nonurgent visits negatively impacts the quality of care and patient safety (prolonged waiting times, delays in diagnosis and treatment, delays in treating seriously ill patients, and medication errors). It also affects the costs of care and patient experience. For hospitals, crowding results in loss of revenue because of patients leaving the ED without being seen, diversion of EDs secondary to patient dissatisfaction, and shifting of the market share to competitors [21]. Moreover, overcrowding exposes ED staff to stressful and unpredictable work-related events, resulting in decreased productivity and increased turnover [22,23].

The body of literature assessing conventional intervention strategies aimed at improving these specific ED issues is highly heterogeneous [24-34]. Proposed interventions vary widely and often require major structural or organizational changes that are not necessarily easily scalable to all hospitals. Importantly, a few address the aforementioned drivers of the ED experience in a scoping and integrative manner along the entire patient journey. Successfully addressing these dimensions requires enlisting patients and families as allies in designing, implementing, and evaluating care systems through patient-centered care approaches [35]. One solution to the serious challenges facing the ED today may be found in information technologies, which have the potential to both reduce institutional burdens and improve patients’ experience [36]. Supported by the rapid spread of mobile devices in the community and their innovative features (eg, versatile connectivity, on-board computing and communication capabilities, privacy, and small size), mobile apps may provide such a solution within the easy reach of end users. However, to date, there is a lack of studies on the potential use of mobile apps to individually support the entire emergency care journey. On the basis of this finding and guided by the principles of patient- and family-centered care [5,35], we developed InfoKids [37], an integrated eHealth solution composed of 3 modules connecting patients, caregivers, and administrative clerks through a web and mobile app, with the aim of supporting the entire emergency care process, thus facilitating caregiving and administrative work and streamlining the arrival of patients in the ED [38]. This system is freely available at Geneva University Hospitals, Geneva, Switzerland, for pediatric patients. It is expected to be soon redesigned to cover the entire population seeking ED care (ie, adult, geriatric, and gynecologic) in a service area of more than 1 million individuals. Before scaling up this app to such a large population, an essential step in determining the potential for the success of this patient-centered eHealth intervention was to assess its capacity to meet end users’ needs and improve health care at our institution before clinical effectiveness testing [39-42].

### Objective

This study aims to evaluate the usability of the InfoKids mobile app to support the entire patient’s ED journey through quantitative and qualitative usability metrics in a laboratory setting. We then aim to identify potential problems related to its use and formulate mitigation measures to inform both the development of its upcoming version as a universal app for all ED outpatient consultations in our hospitals and future mobile app development in this medical field by other research groups.

## Methods

### Study Design

The usability of the app was assessed through a scenario-based, summative evaluation of human-computer interactions using a mixed methods approach [43]. Multitask quantitative and qualitative usability metrics were used and are described in detail in subsequent sections.

### Definition of Usability

Usability is defined as “the extent to which a product can be used by specified users to achieve specified goals with effectiveness, efficiency and satisfaction in a specified context of use” [44]. Usability of a mobile app can be measured by the completeness and successfulness whereby users solve specified tasks centered around the main features of the app. Conversely, systems with poor usability can lead to low goal achievement efficiency or technology not being used [45].

### Participants and Setting

The study was conducted in a medical informatics usability laboratory room at Geneva University Hospitals to standardize the intervention and technically facilitate measurements. The evaluation framework was a user-task-system interaction, deliberately omitting the user’s real environments [43]. Tasks were performed on an LG G5 mobile phone with a 5.3-inch screen size at a resolution of 2560×1440 pixels and an Android operating system V7. According to recommendations on the minimum sample size required to conduct a summative evaluation, at least 15 participants were planned to be recruited [46]. Participants were recruited through advertisements posted on Facebook groups and displayed at the Geneva University Medical Center. Participation was open to adults with children of pediatric age (0-16 years). Exclusion criteria were non–French-speaking persons and those who had previously used the app.

### InfoKids Mobile App

#### Overview

The app was developed by a multidisciplinary team using a user-centered design approach to support each dimension of patient-centered care [37], which is an important approach to consider when developing a mobile health (mHealth) tool for patients. It is primarily defined by considering the needs and values of each patient and helping them to be more actively engaged with shared decision-making about their care [35,47]. Such patient involvement is a key element in high-quality health care [48].

**Figure 1:**
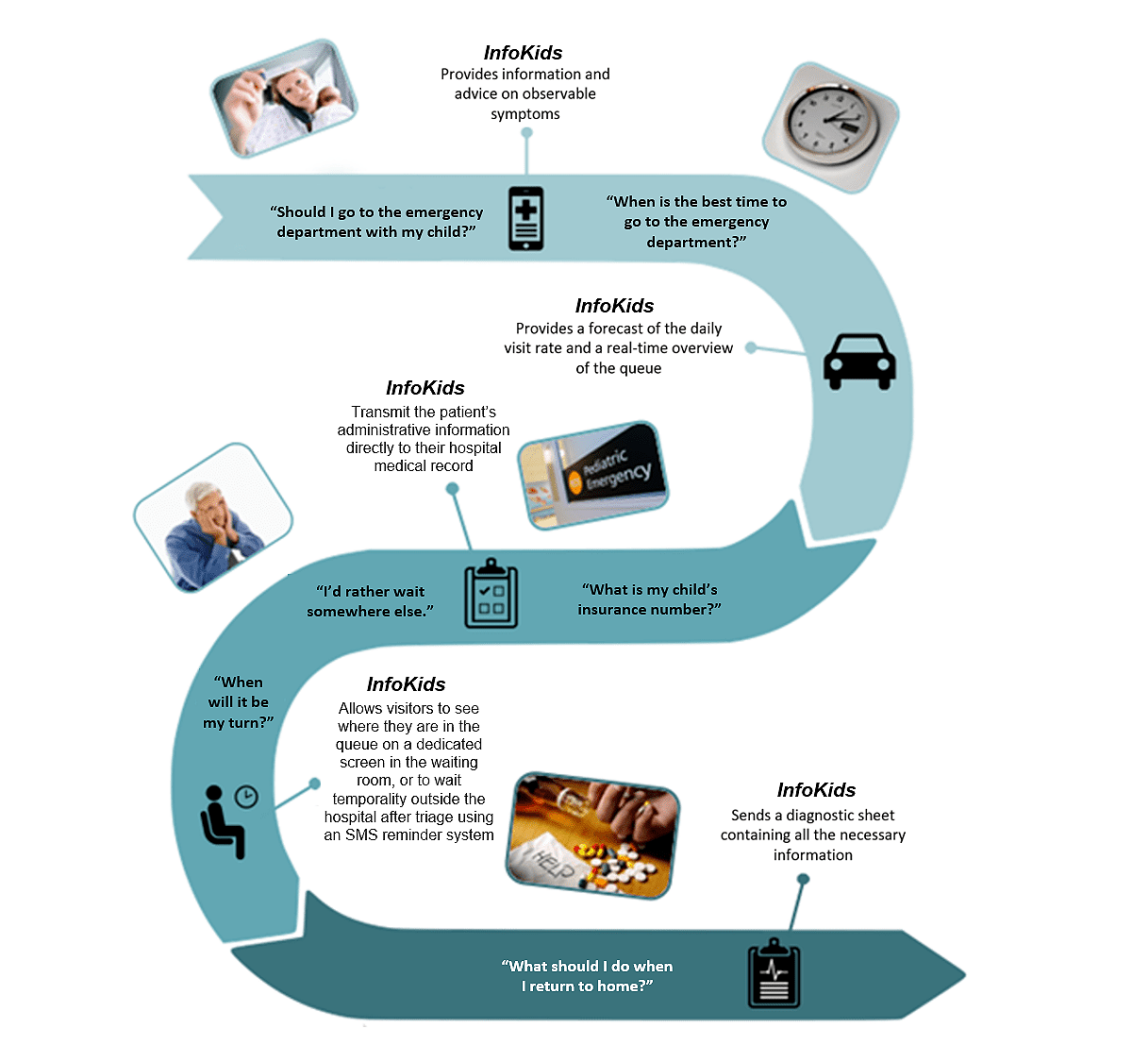
The *InfoKids* app process.

A needs analysis guided by the Picker Institute’s patient-centered care dimensions was conducted among patients and their relatives to identify the specific requirements for the app [49]. System specifications were also identified to translate them into functionalities based on the collected needs of pediatric emergency physicians and nurses and observations of the workflow of caregivers and administrative clerks (Figure 1) [37]. Observations were performed to map out a generic patient journey [37,50]. Improvements were identified from this upstream work and incorporated into the app. In the previous stages of the app’s development, heuristic evaluations were performed by 3 ergonomics experts following the guidelines given by Nielsen and Mack [51] to identify any problems and correct them before proceeding with usability testing. In its current version, the InfoKids mobile app is designed to support parents throughout their entire journey in the pediatric ED; that is, from the onset of the first symptoms to their return home. The interface was designed using hedonic elements to make it more enjoyable and aiming to increase its acceptance. The app is available to the local community through free downloads from the Apple App and Google Play stores.

#### Preconsultation Stage

**Figure 2:**
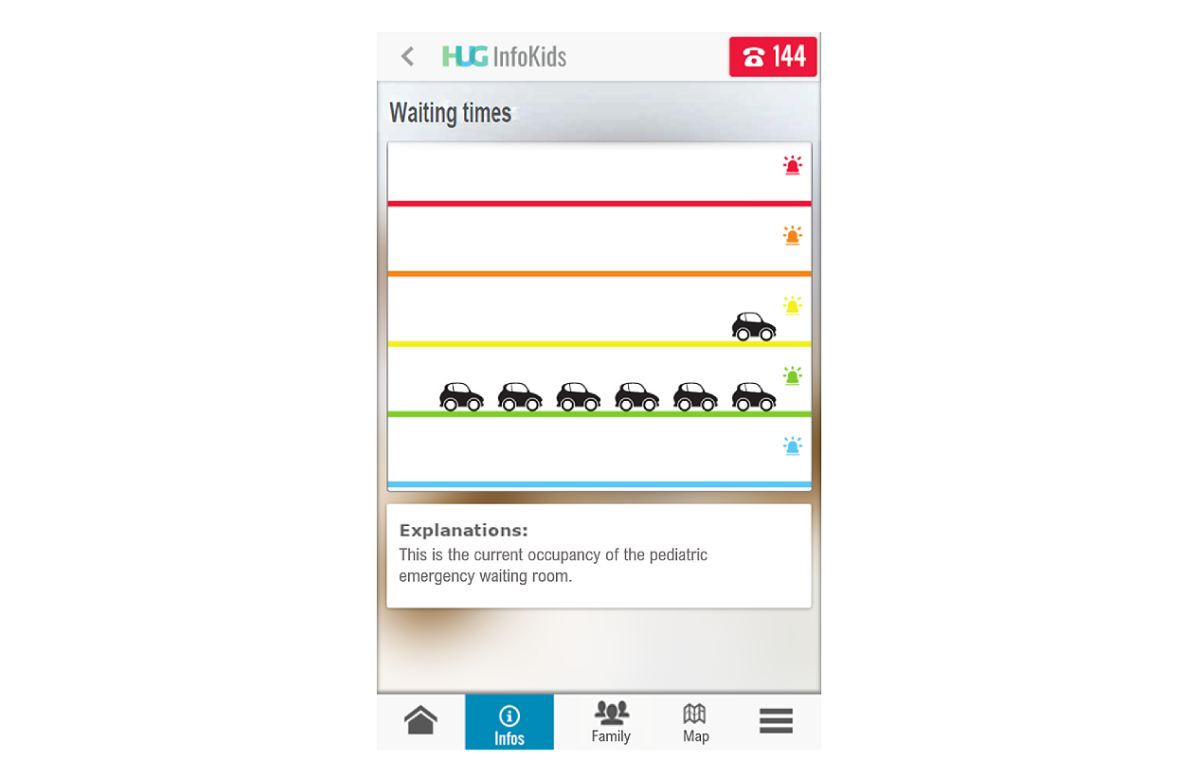
Screenshot of the *InfoKids* mobile app displaying the emergency department occupancy in the waiting room in real time. The Canadian Triage and Acuity Scale categorizes patients by both injury and physiological findings and ranks them by severity from 1 (highest, red) to 5 (blue). By clicking the 144 icon, the user is connected directly to the national emergency call center. HUG: Hôpitaux Universitaires de Genève.

**Figure 3:**
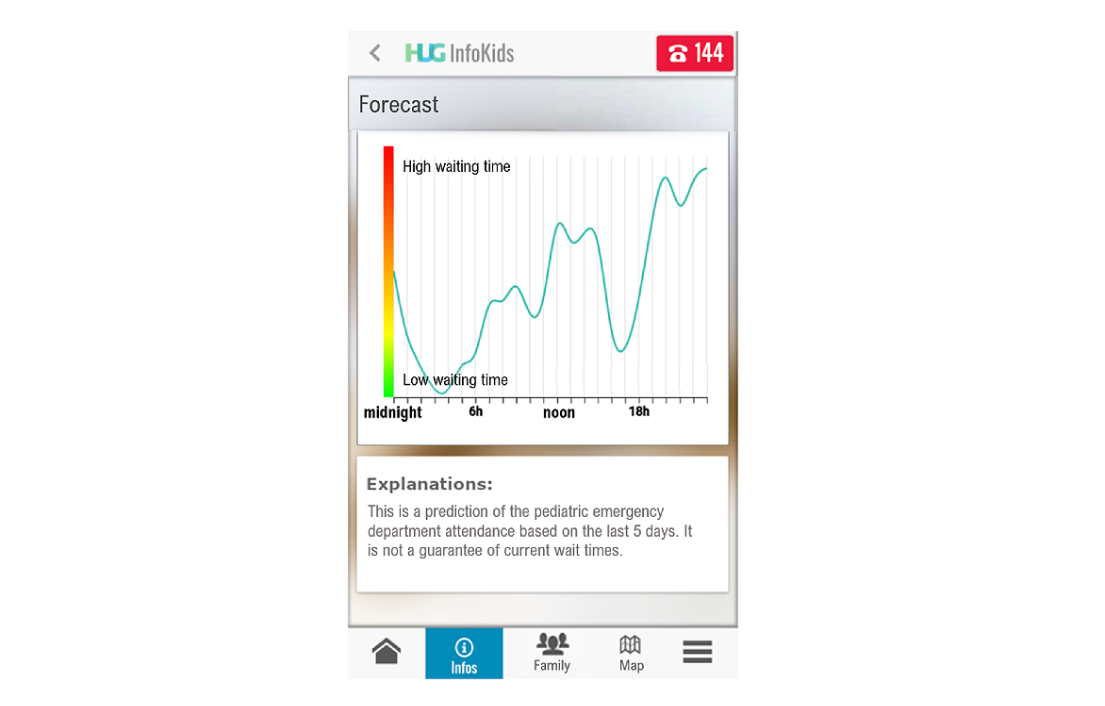
Screenshot of the *InfoKids* mobile app displaying forecasts of daily occupancy based on the statistics of the previous 5 days. The vertical graduation from green (bottom) to red (top) indicates the expected daily occupancy rate from low to high. HUG: Hôpitaux Universitaires de Genève.

The app guides parents through a hierarchical organization of symptoms with medical advice on actions to take; that is, manage the symptom at home, need to visit a private practitioner, or require an ED visit. Classification of symptom terminology was established through a card-sorting study [52]. First, this allows parents to make better decisions on how to deal with symptoms and decide whether to consult. Second, the app contains educational videos aimed at responding to the most common questions that parents may have when visiting the ED. Third, it emotionally supports patients by avoiding unrealistic expectations through the display of ED waiting room occupancy in real time. Occupancy is represented by a metaphoric display of a road where patients are represented as cars queuing (Figure 2). According to the Canadian Triage and Acuity Scale [53], 5 levels of emergency are represented by 5 lanes, as displayed on the screen. Each patient is represented by a car in the sequential order of arrival from right to left for each lane, left being the most recent arrivals. Patients with the highest level of urgency are represented by an ambulance rather than a car. Notably, the same view is displayed on a large television screen hanging on the wall of the ED waiting room. The app also provides a graphic forecast of daily occupancy based on statistics from the 5 previous days. This allows a better distribution of visits throughout the day by offering patients the possibility to consult during the least busy periods (Figure 3) and better perceive expected wait times before being seen by a physician. The app also provides guidance on the hospital location through GPS features and informs the hospital in real time of the patient’s upcoming arrival.

#### Per-Consultation Stage

When parents decide to consult, they can inform the ED of their arrival by a simple click. By doing so, administrative entries recorded in advance within the app are automatically and securely communicated to the hospital. This aims to empower the patient as warrantor of the quality of the administrative data stored in the clinical information system and to reduce the risk of patient misidentification [54]. It also aims to improve the efficacy of ED organizations by shifting the paradigm from an impromptu influx of patients arriving *at the door* to an anticipated occupation, allowing a more efficient management of medical resources. In addition, after triage and when appropriate, patients with nonurgent conditions are offered the possibility to leave the ED temporarily without losing their position in the waiting queue and then called by semiautomated phone messages as soon as a physician is available. These features enable the hospital to act upstream for the regulation of patient flow and overcrowding by a more judicious allocation of health care resources, such as a more rational repartition of caregivers and consultation rooms.

#### Postconsultation Stage

At the time of discharge, the app automatically sends an informative sheet based on the patient’s diagnosis, thus assuring a personalized follow-up. Each sheet offers clear explanations regarding the current condition or trauma, appropriate treatment, prerequisites for a return to the community, and symptoms that require medical attention. The quality and safety of the information provided rely on the core information library supplied by pediatric emergency physicians and endorsed by Geneva University Hospitals. All these features (Table 1) are explained in an in-app tutorial composed of pop-ups and videos.

**Table 1:** Summary of InfoKids functionalities per stage of consultation.

Stage	Functionality	Goal	User actions
Preconsultation	Creation of a user profile	Share securely patient information with the hospital.	Enter parent and child legal information (identity, postal address, insurance, etc) and health records.
Preconsultation	Tutorial	Inform how to use the app and how a consultation at the ED^a^ takes place.	Browse the tutorial.
Preconsultation	Real-time visualization of ED waiting room occupancy	Assist in making decisions about the most appropriate time to consult at the ED.	Visualize occupancy and forecasts.
Preconsultation	Symptoms decision tree classifier	Help with the decision to consult and improve the patient experience.	Identify the symptoms and obtain advice on how to manage them.
Preconsultation	Guidance	Find the ED location (GPS).	Follow the GPS.
Per-consultation	ED already informed upon patient arrival	Anticipate the patient’s arrival.	Confirm departure.
Per-consultation	Symptoms, chronic illnesses, allergies, and usual treatments entered by the parent into the app are automatically communicated to the ED	Empower patient as warrantor of the quality of the administrative data stored in the clinical information system; reduce patient misidentification.	Enter the child’s administrative and personal data in the app beforehand; automated sending of this information at the time of announcement of departure to the ED by a simple click.
Per-consultation	Temporarily leave the ED while waiting for a scheduled consultation	Reduce the waiting time and improve the patient experience.	Accept the legal discharge document, allowing to temporarily leave the ED.
Postconsultation	Personalized diagnostic sheet	Improve therapeutic adherence and the patient experience.	Provide access to diagnostic and therapeutic follow-up.

^a^ED: emergency department.

### Procedure

Participants were invited by emails to individual sessions. The study procedure was explained to the participants upon arrival at the evaluation laboratory. Written informed consent was obtained from all the participants. After completing a baseline questionnaire on demographics and user experience with smartphones, the participants were asked to imagine themselves in a situation where they had heard about the InfoKids app and to follow a scripted and timed standardized scenario. The scenario was developed to sequentially guide the user toward the completion of 9 goal-oriented tasks covering the main functionalities of the app (Textbox 1; Multimedia Appendix 1). The sequence of tasks reflected the sequence of actions that parents seeking medical advice for their sick child with worrying symptoms at home would have to perform. For reasons related to the study design and use of the app, the possibility of patients being able to temporarily leave the ED while waiting for a scheduled consultation was not evaluated but will be the subject of further research. For greater realism, the dates and times were adapted to the time of the experiment. No training on the app was offered before the evaluation began to avoid preparation bias. The participants were not given any assistance to complete the tasks. Study investigators only intervened to encourage participants to keep talking during the intervention, thus avoiding bias of results and minimizing any disruption of participants’ thoughts. The participants were informed that their interaction with the app and their verbal exchanges would be video recorded.

**Goal-oriented test tasks.**

**Goal-oriented test tasks**

- Task 1: open the app, enter your personal data as requested, and accept the terms of use.
- Task 2: create a profile for your child and close the app (Multimedia Appendices 2 and 3).
- Task 3: imagine that 2 days later, your child has cough and you are seeking medical advice. Open the app and look for advice (Multimedia Appendix 4). Read the tips on what you can do at home to manage the situation on your own. Also read the tips on when you should go to the pediatrician in the next 24 hours. Close the app.
- Task 4a: 1 week later, you plan to go to the pediatric emergency room because of the worsening of your child’s cough and health condition. You are wondering about the current emergency room occupancy and want to see how busy the waiting room is (Figure 2). The date is (date of examination), current time is (time of examination). Are there many people in the emergency department (ED)? Can you describe what the cars represent on the screen? Can you describe what the different lines represent?
- Task 4b: Does occupancy in the ED over the last few days allow you to predict whether the wait on that day will be long? Can you describe what the graph represents (Figure 3)?
- Task 5: you decide to go to the ED with your child. Inform the ED of your arrival and return to the home page (Multimedia Appendix 5).
- Task 6a: you are seeking information on the location of the ED. Go to the tutorial to find information on how to use the mapping tool (GPS).
- Task 6b: after viewing the tutorial, indicate the location of the ED building on the map and return to the home page (Multimedia Appendix 6).
- Task 7: you went to the emergency room and came home. You receive a notification on your app regarding the diagnosis made in the ED and read it. What is the physician’s diagnosis? What home care information is necessary?

To understand participants’ thoughts, the concurrent *think-aloud* method was applied by asking them to verbalize during task completion [55]. Upon completion of the scenario, the moderator had a debriefing with each participant following a semistructured grid interview, with the aim of assessing overall experience with the tool and usability improvements and perform a retrospective *think-aloud* method to analyze difficulties encountered and understand their causes [56]. Finally, to assess user satisfaction, the participants were asked to complete the System Usability Scale (SUS) questionnaire [57,58].

### Scenario

A pediatric emergency physician (JNS) wrote a credible and standardized scenario based on these tasks, which was then screened and approved by two ergonomists (JR and AR) at the evaluation laboratory. In the scenario, the participant decides to install the app in the eventuality that an ED visit might be necessary. Shortly after, the participant (ie, the parent) needs to use the app for the first time following the onset of cough in their child. A week later, when the cough and the child’s health had deteriorated, the parent had to use the app again to be guided and supported to go to the ED with the child.

### Usability Analysis

#### Quantitative Evaluation

The participant’s task performance was measured by the following metrics:

1. *Effectiveness* is defined as the accuracy and completeness in which users achieve the specified goals [44]. Effectiveness is calculated in three different ways:
  - *Task completion rate (TCR) per participant*, that is, the percentage of tasks successfully completed, whether with ease or difficulty [59]. This is calculated using the following equation:
    TCR per participant = (number of tasks completed successfully / total number of tasks undertaken) × 100 **(1)**
    When a task cannot be started and evaluated (ie, because of a problem with the Wi-Fi connection), it is coded as *nonavailable*.
  - *TCR per task*, that is, the percentage of participants who successfully completed a given task, whether with ease or difficulty [59]. This is calculated using the following equation:
    TCR per task = (number of participants who completed successfully / total number of participants) × 100 **(2)**
    When a task cannot be started and evaluated (ie, because of a problem with the Wi-Fi connection), it is coded as *nonavailable*.
  - *Distribution of task success by task* is defined as the proportion of participants completing a task according to three possible levels of achievement: (1) the task is considered *completed with ease* when the user has successfully completed the task without any errors or difficulties; (2) *completed with difficulty* when the task was completed, but with difficulties that could have been solved by the participant; and (3) *failed to complete* when the task is left incomplete or abandoned or the participant gave incorrect answers. When a task cannot be started and evaluated (ie, because of a problem with the Wi-Fi connection), it is coded as *nonavailable*.
2. *Efficiency* is defined as the level of resource use required for users to achieve specified goals in relation to accuracy and completeness [44]. This is calculated in three different ways:
  - *Time on task* is defined as the average amount of time (in seconds) taken to complete a given task from the moment the participant finished reading the instructions until the task was completed (whether with ease or with difficulty) or abandoned.
  - *Time-based efficiency (TBE)* is defined as the time spent by users in absolute value to ensure the accurate and complete achievement of tasks using the 2 equations described in a study by Ben Ramadan et al [59].
  - *Overall relative efficiency (ORE)* is defined as the ratio of the time spent by effective users to ensure accurate and complete achievement of tasks to the total time taken by all users (ie, including the time spent by ineffective users) using the 2 equations described in a study by Ben Ramadan et al [59].
3. *Satisfaction* measured by administering the SUS questionnaire designed by Brooke [57,58], a highly robust and versatile tool to measure participants’ subjective assessment of usability [60]. SUS is a 10-item questionnaire (Multimedia Appendix 7), with 5 response options for respondents for each item, based on their level of agreement from 1 (*strongly disagree*) to 5 (*strongly agree*). Following the Brooke scoring system [57,58], for odd-numbered statements 1, 3, 5, 7, and 9 (positively worded items), the score contribution is equal to the scale position minus 1 (eg, *strongly agree* 5−1=4). For even-numbered statements 2, 4, 6, 8, and 10 (negatively worded items), the score contribution is equal to 5 minus the scale position (eg, *strongly agree* 5–5=0). Each score contribution falls within the range of 0 to 4. The participants’ scores for each item are then summed and multiplied by 2.5 to convert the original scores from 0 to 40 to 0 to 100. Although the scores range from 0 to 100, these are not percentages of usability and should be considered only in terms of their percentile ranking. To obtain an SUS score of 100, the respondent must answer 5 to all odd questions and 1 to all even questions. It is generally considered that a score is good starting from 75 and fair between 50 and 75. A score below 50 reveals strong disagreement in terms of satisfaction [60]. As the participants were French speaking, the French translation of the questionnaire was used [61]. As age could be a potential confounder correlated with usability scores [60], we also analyzed SUS scores according to two age categories (≤40 years and >40 years).

#### Qualitative Evaluation

Qualitative data from the concurrent, retrospective *think-aloud* and debriefing were used to assess the overall experience with the tool, identify usability problems, understand the cause of difficulties, and identify usability improvements. Usability problems encountered by the participants during the tasks were rated using the Nielsen severity scale [62] and categorized using the ergonomic criteria of Bastien and Scapin [63]. The Nielsen scale ranges from 0 to 4, with higher scores positively correlated with greater problems (0=no usability problem; 1=cosmetic problem that does not need to be addressed unless extra time is available on the project; 2=minor usability problem: fixing this should be given low priority; 3=major usability problem: important to fix and should be given high priority; and 4=usability catastrophe: imperative to fix this before releasing the product). The Nielsen criteria [51] used to rate the severity of usability problems are (1) the frequency of occurrence of a problem (common or rare?), (2) its impact on the user’s experience (easy or difficult for users to overcome?), and (3) its persistence (a unique problem on first use or will it persist to bother users?). As some studies have shown that severity ratings are subjective and can vary significantly from one assessor to another [64], they were conducted independently by 2 ergonomists. In case of disagreement, the ratings were averaged [65]. However, to avoid disagreement, both ergonomists agreed to classify usability problems that led to failure as a *usability catastrophe*. The Bastien and Scapin [63] method consists of a list of 18 ergonomic criteria that are generally used to identify and understand the most well-known interface problems. The categorization of usability problems following these criteria was performed independently by both ergonomists. In case of disagreement, the evaluators discussed together to reach a consensus.

### Data Collection

Participants’ task performance was video-recorded and audio-recorded to retrospectively analyze the usability of the app. Video and audio captures were acquired with an Elmo L-l2iD camera document placed above the phone. Morae software (TechSmith Corporation) was used to analyze the video and audio recordings of participants’ interactions with the app. Subsequently, the recordings and usability metrics were transcribed onto Microsoft Excel spreadsheets. The SUS paper questionnaires were collected immediately after the intervention and subsequently transcribed onto Microsoft Excel spreadsheets. Two researchers (JR and VGR) analyzed the success rates of each task and their duration independently of each other. In case of disagreement, both researchers discussed together to reach a consensus. All data collected were anonymized.

### Data Analysis

Descriptive statistics were used to summarize continuous measures at a significance level of .05. Frequency counts were used for summarizing categorical measures. Age categories and SUS mean scores were compared to make comparisons between user characteristics and satisfaction. Data were analyzed, and graph figures were created with GraphPad Prism 9 and Microsoft Excel.

### Ethics Approval and Consent to Participate

The study was submitted to the Regional Research Ethics Committee (Req-2021-00505), which waived the need for further evaluation by issuing a *no objection* statement, as such projects did not fall within the scope of the Swiss federal law on human research [66]. Only data from a fictitious patient were used in this study. Written informed consent was obtained from all participants before the intervention. No participants’ medical information was used. Participants were not identifiable on video and audio recordings. Participants’ data and results obtained through the intervention were deidentified and assigned an individual identifying code that did not contain identifying information. Data were secured by protected access passwords at Geneva University Hospitals on secured hard disks. This study was conducted in accordance with the Declaration of Helsinki [50] and principles of Good Clinical Practice [51].

## Results

### Participant Characteristics

Between June and September 2017, a total of 17 participants participated in the study. Baseline demographic characteristics are shown in Table 2.

**Table 2:** Demographic characteristics of study participants (N=17).

Characteristics		Values, n (%)
**Gender**		
	Woman	15 (88)
	Man	2 (12)
**Age categories (years)**		
	21-30	3 (18)
	31-40	4 (24)
	41-50	8 (47)
	51-60	2 (12)
**Number of children**		
	1	9 (53)
	2	5 (29)
	3	2 (12)
	4	1 (6)
**Parents with a child aged (years)**		
	0-3	6 (35)
	3-6	7 (41)
	6-9	7 (41)
	9-12	1 (6)
	12-15	3 (18)
**Already visited Geneva pediatric ED^a^**		
	Yes	12 (71)
	No	5 (29)
**Type of phone**		
	iOS	7 (41)
	Android	9 (53)
	Windows phone	1 (6)
**Possession of a smartphone**		
	<1 year	0
	From 1 to 2 years	0
	More than 2 years	17 (100)
**Frequency of mobile apps use**		
	Often (daily)	17 (100)
	Regularly (several times per week)	0
	Sometimes (once to several times per month)	0
	Rarely (once to several times per year)	0
	Never	0

^a^ED: emergency department.

### Quantitative Evaluation

#### Effectiveness Per Participant

**Figure 4:**
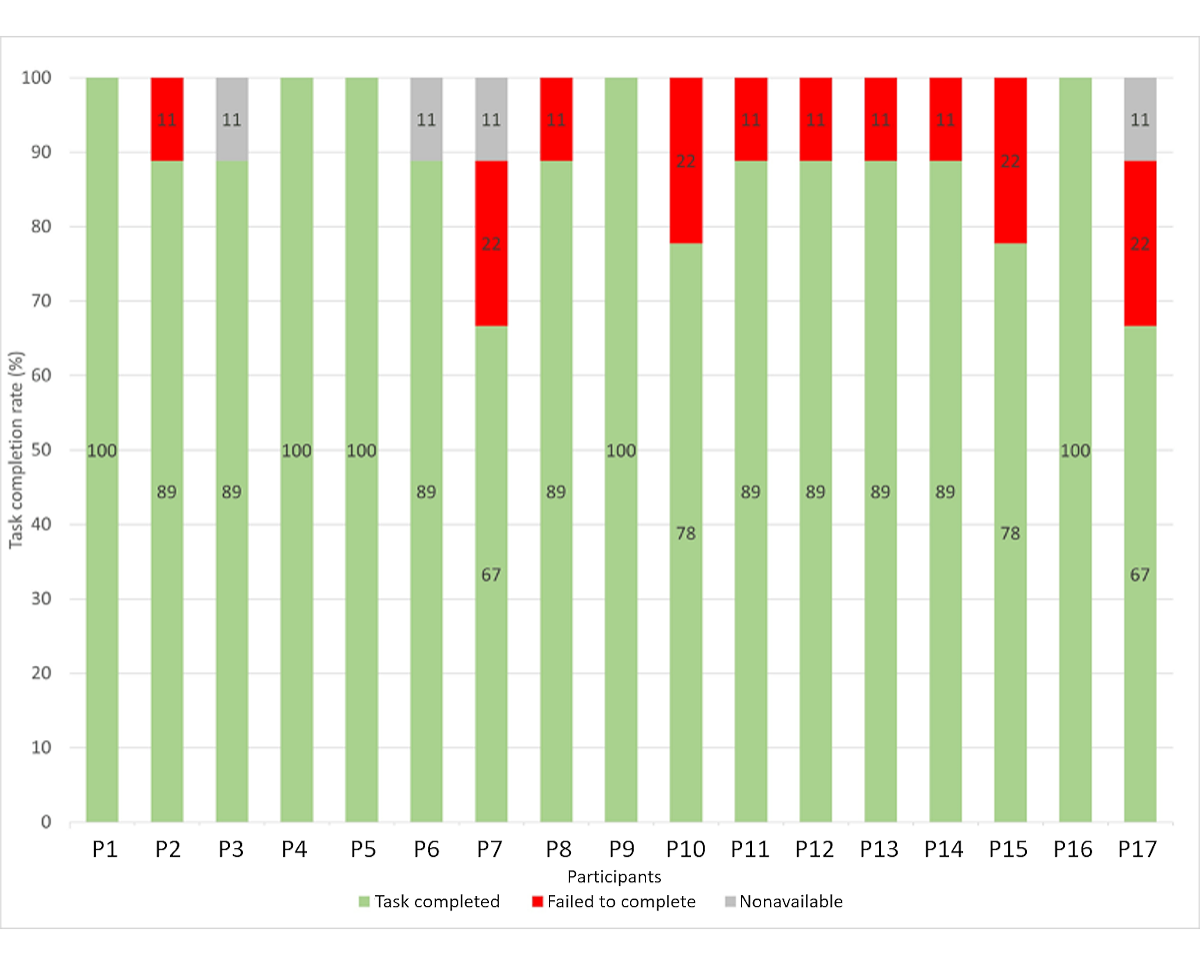
Task completion rate per participant for the 9 assigned tasks. *Task completed* represents the percentage of tasks successfully completed by a participant, whether with ease or difficulty. *Failed to complete* defines the percentage of tasks that participants failed to complete. *Nonavailable* represents the percentage of missing data when a task could not be started and evaluated.

The overall completion rate (tasks completed and failed) was 88.2% (135/153). A total of 4 participants did not perform some tasks, 2 (50%) participants ignored task 6a, 1 (25%) participant experienced a problem with the Wi-Fi connection in task 6b, and 1 (25%) participant experienced a software bug in task 7. The mean overall success rate, defined as the percentage of tasks that participants completed successfully (whether with ease or difficulty), was 88.2% (135/153; SD 10.63%; 95% CI 83%-93%). An analysis of almost 1200 usability tasks showed that the minimum accepted average TCR was 78% [67]. In this study, the TCR per participant ranged from 67% to 100% (Figure 4).

#### Effectiveness Per Task

**Figure 5:**
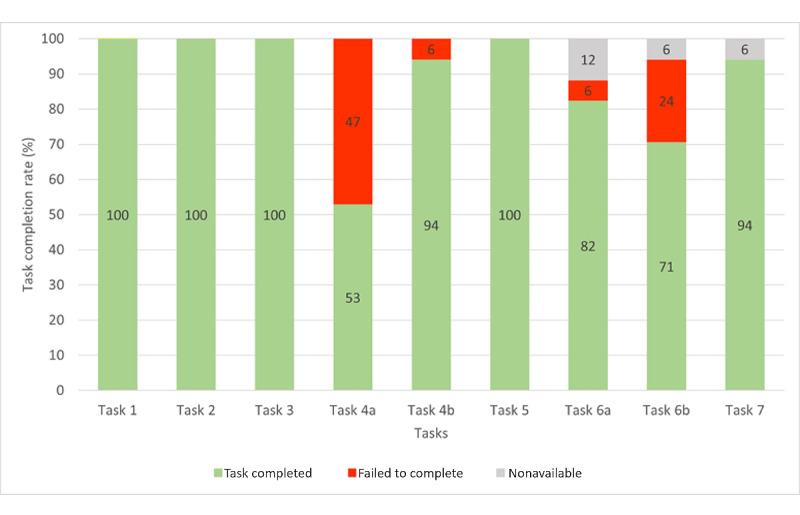
Task completion rate per task (N=17 participants). *Task completed* represents the percentage of participants who successfully completed the task, whether with ease or difficulty. *Failed to complete* defines the percentage of participants who failed to complete the task. *Nonavailable* represents the percentage of missing data when a task could not be started and evaluated.

Of the 9 assigned tasks, 4 (44%) were achieved 100% by all participants (Figure 5); 2 (22%; tasks 4b and 7) reached a TCR per task of 94%; 1 (11%; task 6a) reached a TCR of 82%; and 2 (22%) scored below 78%: task 6b with a value of 71% and task 4a with a value of 53%. Of note, all tasks with a value of less than 100% were related to either browsing through the pages of the app or understanding the information displayed. Figure 5 shows that task 4a appeared to be most complicated. Tasks 4b, 6a, and 6b also seemed problematic for some participants.

#### Task Success Distribution Per Task

**Figure 6:**
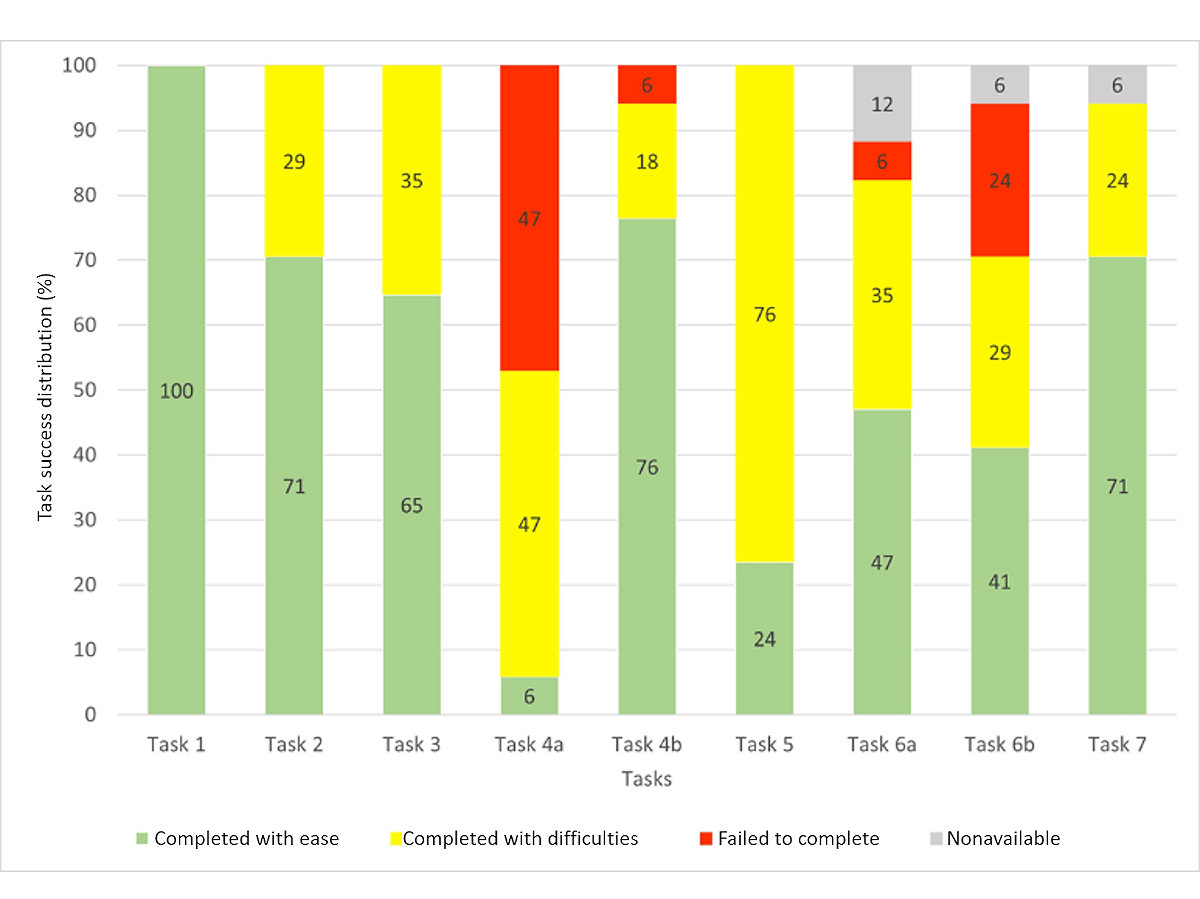
Task success distribution per task (N=17 participants). *Completed with ease* represents the percentage of participants who completed the task with ease. *Completed with difficulty* represents the percentage of participants who completed the task with difficulties. *Failed to complete* defines the percentage of participants who failed to complete the task. *Nonavailable* represents the percentage of missing data when a task could not be started and evaluated.

The observed task success distribution is shown in Figure 6. Task 1 was completed with ease by all the participants (17/17, 100%), followed by task 4b (13/17, 76%). Tasks 2 and 7 were completed with ease by 71% (12/17) of the participants. Task 3 was completed with ease by 65% (11/17) of the participants, but tasks 6a, 6b, 5, and 4a were completed with ease by only 47% (8/17), 41% (7/17), 24% (4/17), and 6% (1/17) of the participants, respectively. Apart from task 1, all tasks led to difficulties with a *completed with difficulties* rate ranging from 18% to 76%. Participants encountered failures during four tasks (4a, 4b, 6a, and 6b), with a *failed to complete* rate ranging from 6% to 47%.

### Efficiency: Time on Task

The mean overall time on task for all tasks was 101.26 (SD 44.07) seconds. Tasks 1, 2, and 4a had a higher time on task than the other tasks (Table 3). These findings showed that the most complicated task (ie, task 4a) was the third most time-consuming task, although it did not require much action compared with tasks 1 and 2, which were the longest and required several pieces of data to be entered into the app, thus explaining their duration.

**Table 3:** Time on task per study task.

Task	Time on task (seconds), mean (SD)
Task 1: Create a parental account	142.58 (38.96)
Task 2: Create a child profile	182.56 (58.64)
Task 3: Find the *symptoms* page	96.86 (46.36)
Task 4a: Find and understand the *waiting times* page	138.61 (71.4)
Task 4b: Find and understand the *forecast* page	55.93 (34.27)
Task 5: Inform of the departure to ED^a^	80.08 (58.98)
Task 6a: Find the *tutorial* page	59.93 (27.06)
Task 6b: Find the *map* page	62.34 (47.16)
Task 7: Find the *diagnostic* sheet	92.46 (52.84)

^a^ED: emergency department.

Multimedia Appendices 8 and 9 show the TBE and ORE for every single performed task, respectively. Multimedia Appendix 8 shows that tasks 1, 2, and 4a had the shortest TBE. Therefore, creating the parental account and the child’s profile was not the most efficient task. Task 4a showed the lowest efficiency, with the shortest TBE (0.0065 tasks per second) and lowest ORE (50.2%).

### Satisfaction: SUS Questionnaire

**Figure 7:**
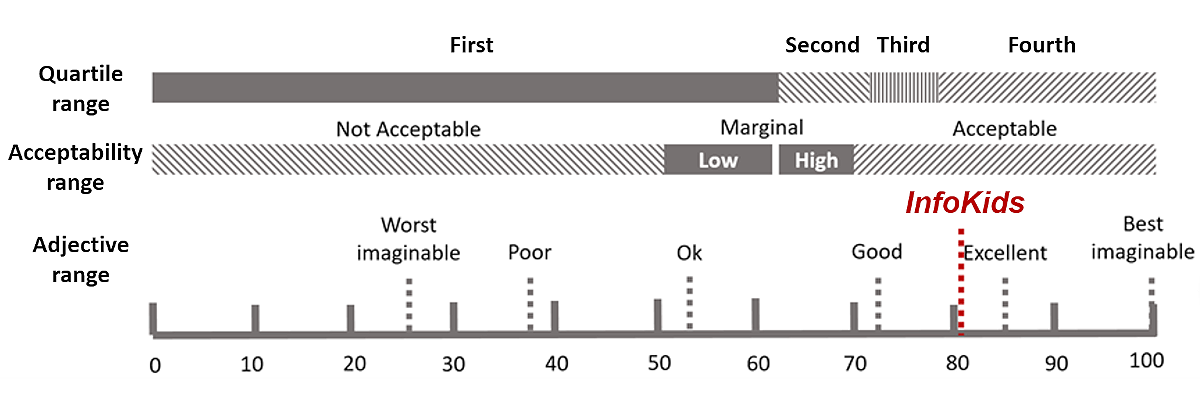
Overview of the modified System Usability Scale rating table with inserted value ranges [68].

The mean overall SUS score was 80.88 (SD 8.57; Table 4). This shows that the usability of the InfoKids app was perceived as good to excellent [68] (Figure 7). The detailed scores indicate that of the 17 participants, 4 (24%) assessed the app as fair, 5 (29%) as good, and 8 (47%) as excellent. Mean SUS scores were similar when analyzed by two age categories, ≤40 years (mean 82.14, SD 9.94 years) and >40 years (mean 80, SD 8.42 years; Mann–Whitney *U* test=29.5; *P*=.60).

**Table 4:** System Usability Scale (SUS) questionnaire results.

	Question 1	Question 2	Question 3	Question 4	Question 5	Question 6	Question 7	Question 8	Question 9	Question 10	SUS score (sum×2.5; maximum 100)
P1	3	3	3	4	4	4	4	4	4	4	92.5
P2	3	3	4	4	3	4	3	4	4	3	87.5
P3	3	2	2	4	3	3	3	2	3	3	70
P4	4	3	3	4	3	3	4	4	3	4	87.5
P5	3	3	3	4	3	2	3	4	3	2	75
P6	3	3	3	4	3	2	3	4	3	4	80
P7	3	1	2	4	3	3	3	4	3	4	75
P8	4	3	3	4	4	4	3	4	4	3	90
P9	3	3	4	4	3	3	4	4	4	3	87.5
P10	4	0	4	0	3	1	4	4	3	3	65
P11	4	3	3	4	3	4	3	4	4	4	90
P12	3	3	3	2	3	3	4	4	4	3	80
P13	4	3	3	3	4	3	4	4	4	4	90
P14	4	3	3	2	3	3	3	4	3	3	77.5
P15	4	3	3	4	4	4	4	4	4	1	87.5
P16	3	3	3	3	3	3	3	2	2	3	70
P17	2	3	3	3	3	3	3	2	3	3	70
Values, mean (SD)	3.35 (0.59)	2.65 (0.84)	3.06 (0.54)	3.35 (1.08)	3.24 (0.42)	3.06 (0.8)	3.41 (0.49)	3.65 (0.76)	3.41 (0.6)	3.18 (0.78)	80.88 (8.57)
Values, median (IQR)	3 (3-4)	3 (3-3)	3 (3-3)	4 (3-4)	3 (3-3)	3 (3-4)	3 (3-4)	4 (4-4)	3 (3-4)	3 (3-4)	80 (75-87.5)

### Qualitative Evaluation

#### Usability Problems

The *think-aloud* method identified 14 usability problems with a total of 81 occurrences. Table 5 describes the frequency of usability problems per task and the frequency of each usability problem that led to task completion with difficulties or failures. A total of 9 usability problems led to difficulties to complete a task only, and 5 led to difficulties to complete a task and failures.

**Table 5:** Frequency of 14 usability problems, difficulties, and failure.

Tasks and usability problems			Frequency of the usability problem (n=81), n (%)		Frequency with which it led to task completion with difficulty (n=62), n (%)		Frequency with which it led to failure to complete the task (n=19), n (%)
**Task 1: create a parental account**							
	None	0 (0)		0 (0)		0 (0)	
**Task 2: create a child profile**							
	Participants expected to access the child’s profile by clicking directly on the card	1 (1)		1 (2)		0 (0)	
	Participants wondered if information had been properly saved	4 (5)		4 (6)		0 (0)	
**Task 3: find the *symptoms* page**							
	Participants did not directly find the symptoms’ list	5 (6)		5 (8)		0 (0)	
	Participant did not directly find the *cough* symptom	1 (1)		1 (2)		0 (0)	
**Task 4a: find and understand the *waiting times* page**							
	Participants did not directly find the *waiting times* page	13 (16)		8 (13)		5 (26)	
	Participants faced difficulties to understand the meaning of the cars and the different colored lines	10 (12)		2 (3)		8 (42)	
**Task 4b: find and understand the *forecast* page**							
	Participants had difficulties in finding the page.	4 (5)		3 (5)		1 (5)	
**Task 5: inform of the departure to the ED^a^**							
	Participants had difficulties in finding this feature.	7 (9)		7 (11)		0 (0)	
	Participants did not understand that they had to select the child.	13 (16)		13 (21)		0 (0)	
**Task 6a: find the *map tutorial* page**							
	Participants expected to access the *map tutorial* directly in the *map* page.	6 (7)		5 (8)		1 (5)	
	Participants had difficulties in finding the *map tutorial* because of a pop-up hiding the button.	2 (2)		2 (3)		0 (0)	
**Task 6b: find the location of the ED**							
	Participants did not understand the meaning of the “H” icon indicating the location of the ED on the map.	9 (11)		5 (8)		4 (21)	
**Task 7: find the *diagnostic* sheet**							
	Participants did not directly find the page.	4 (5)		4 (6)		0 (0)	
	Participants had difficulties in finding the section to access the *diagnostic* sheet.	2 (2)		2 (3)		0 (0)	

^a^ED: emergency department.

Identified usability problems were rated by their severity scores. Of the 81 occurrences of usability problems, 2 (2%) were rated with a severity score of 1 (cosmetic), 22 (27%) were rated 2 (minor), 17 (21%) were rated 2.5 (between minor and major), 11 (14%) were rated 3 (major), and 29 (36%) were rated 4 (catastrophic; Table 6; Multimedia Appendix 10 [62,63]). None of the participants experienced major or catastrophic usability problems when completing tasks 1, 3, and 7 but tasks 2, 4a, 4b, 5, 6a, and 6b were the most problematic. When analyzing the time on task, the longest time taken to complete task 2 seemed to be related to the time required to access and fill this page compared with other tasks, although the completion rate was optimal and usability problems were reported as minor. The third longest time taken to complete task 4a appeared to be related to the many usability problems graded as catastrophic.

**Table 6:** Severity scores, identification of usability problems, frequency, percentage, and related task.^a^

Usability problems		Value (n=81), n (%)	Related task
**Severity score 1**		2 (2)	N/A^b^
	Access the child’s profile	1 (1)	2
	Find the cough symptom	1 (1)	3
**Severity score 2**		22 (27)	N/A
	Select the child	13 (16)	5
	Message hiding the button to access the map tutorial	2 (2)	6a
	Find the symptom page	5 (6)	3
	Find the diagnostic sheet: Select the history section	2 (2)	7
**Severity score 2.5**		17 (21)	N/A
	Find the waiting times page	13 (16)	4a
	Find the diagnostic sheet: Reach the information page	4 (5)	7
**Severity score 3**		11 (14)	N/A
	Record the information entered	4 (5)	2
	Find the page to inform about departure to the ED^c^	7 (9)	5
**Severity score 4**		29 (36)	N/A
	Find the forecast page	4 (5)	4b
	Find the location of the ED	9 (11)	6b
	Understand the waiting times page	10 (12)	4a
	Find the map tutorial	6 (7)	6a

^a^Severity score: 1=cosmetic, 2=minor, 3=major, and 4=catastrophic.

^b^N/A: not applicable.

^c^ED: emergency department.

Most problems identified (34/81, 42%) were related to the significance of codes’ criteria, whereas 35% (28/81) problems were related to compatibility criteria, 21% (17/81) to the guidance criterion, and 2% (2/81) to explicit control (Table 7).

**Table 7:** Ergonomic criteria associated with identified usability problems with its number of occurrence and frequency.

Ergonomic criteria		Usability problems	Number of occurrence and percentage of the usability problem, n (%)	
**Guidance**				17 (21)
	Guidance—prompting	Select the child	13 (16)	
	Guidance—immediate feedback	Recording of information entered	4 (5)	
**Explicit control**				2 (2)
	User control	Message hiding the button to access the *map tutorial*	2 (2)	
**Significance of codes**				34 (42)
		Find the *symptom* page	5 (6)	
		Find the *cough* symptom	1 (1)	
		Find the *waiting times* page	13 (16)	
		Find the *forecast* page	4 (5)	
		Find the location of the ED^a^	9 (11)	
		Find the *diagnostic* sheet: select the *history* section	2 (2)	
**Compatibility**				28 (35)
		Access the child’s profile	1 (1)	
		Understand the *waiting times* page	10 (12)	
		Find the page to inform about departure to the ED	7 (9)	
		Find the *map tutorial*	6 (7)	
		Find the *diagnostic* sheet: reach the information page	4 (5)	

^a^ED: emergency department.

#### Debriefing Interviews

All participants (17/17, 100%) reported positive feedback regarding their overall experience with the app. More specifically, when asking them about the strengths of the app by an open question, 71% (12/17) of participants emphasized the usefulness of the proposed features, such as the information on waiting times, advice according to symptoms, the diagnostic sheet, and the ability to inform the ED of their arrival. Moreover, 65% (11/17) noted the ease of use because of the quickly accessible menu and its intuitiveness.

Regarding app improvements and mitigation measures, 35% (6/17) of participants expressed several needs: (1) an improved ED geolocalization on the map; (2) rewording the *history* section to *diagnostic history* to find the sheet more easily; (3) improved explanation of the meaning of the 5 colored emergency lanes; and (4) placement of the *I am coming to the ED* button on the home page to facilitate its access. Participants also expressed their wish to have new features such as information about the laboratory results and treatment plan in the *diagnostic* sheet (3/17, 18%), ability to exchange with the ED directly through the app with a *chat* option (1/17, 6%), and the ability to share the *diagnostic* sheet with another family member (1/17, 6%).

## Discussion

### Principal Findings

In this study, we report an overall good-to-excellent perceived usability of a patient-centered mHealth app aimed at covering the entire emergency care process by supporting patients before, during, and after an ED visit. Given the high percentage of patient-centered assigned tasks that participants successfully completed, we observed a good overall rate of understanding of how the app worked. Participants found most of the features useful, particularly the recommendations provided according to their child’s symptoms, access to information related to waiting times and the diagnosis made in the ED, and ability to inform the ED upon their arrival. However, the ergonomic evaluation identified 81 occurrences of 14 usability problems, of which 50% (7/14) were serious, as their severity ratings were either major or catastrophic. These results indicated areas for app improvements. From participants’ and ergonomists’ suggested usability improvements, mitigation measures were listed to further improve the app and avoid barriers to its adoption (Table 8).

**Table 8:** Identified usability problems and mitigation measures.

App’s features and identified usability problems			Mitigation measures
**Editable list of children**			
	The edit button on the child’s profile was not obvious enough	The whole patient’s profile card should be made clickable.	
**Child’s profile page**			
	Uncertainty as to whether the entries for chronic illnesses and regular medications are saved in the app	Entries for chronic conditions and regular medications should be visible on the patient’s profile page.	
**Browsing through the pages or menus**			
	Difficulty in locating the ED^a^ departure announcement button	The *I am coming to the ED* button should also be placed on the home page.	
	Difficulty in locating the *diagnostic* sheet	The *history* page should be changed to *diagnostic history*.	
	Difficulty in locating the *map tutorial*	The *map tutorial* should be placed directly in the *map* page. The tutorial could start automatically when the map is used for the first time, as is the case in many apps.	
	Difficulty in locating the *waiting times* page, the *forecast* page, and the *symptom* page	The tree-testing and card-sorting techniques should be used to improve the information architecture and the nomenclature. A search bar should also be added.	
**Symptoms’ decision tree**			
	Difficulty in browsing through the symptom’s decision tree	A search bar and more redundancy should be added.	
**Real-time display of the ED waiting room**			
	The meaning of “occupancy” in the waiting room was not clear for nonacquainted users	The busy screen should be redesigned using a more explicit graphic representation and adding a caption. Representing patients by avatars and not by cars could be more intuitive for the user.	
**Geolocation and guidance to the ED**			
	Geolocation markers were not explicit enough on the *map* page	Knowing that icons are images and that images can be polysemic, their understanding can vary from one person to another. To reduce this effect, a locator pin with a textual indication could be used. In addition, it could be enlarged and bounced to attract the user’s attention.	
**ED departure feature *I am coming to the ED***			
	No prompt to indicate to the user that they must select the child to be announced on departure to the ED	A selection checkbox should be set up so that users understand that they need to select a child.	
	It should be easily possible to hide the pop-up message confirming the patient’s departure to the ED	The chevron must be enlarged to make it more visible.	

^a^ED: emergency department.

Apps’ attrition has emerged as an area of particular concern in recent literature on new technological innovations [69,70]. Even when apps are evidence based, this does not guarantee that they will be used consistently over time. Similar to other health information technologies, the benefits of apps can only be achieved if end users intend to adopt them [71]. Poor usability and a lack of user-centered design have been described as 2 drivers for low adoption rates of mobile apps [45]. Although usability has been identified as a key component of good practice in the development of digital apps [72], only a small fraction of medical apps publish their usability evaluation results, despite their growing number [42]. The main concerns of these apps are health conditions or diseases such as mental health [45,73], cancer [74,75], nutrition [76], diabetes [77,78], chronic disease self-management [79,80], and child health [81-86], among others [42].

However, there is no app that addresses more broadly patients’ accompaniment throughout their entire ED care journey (ie, before, during, and after their visits), as well as providing personalized health information and support to manage illness or trauma. We found only 2 studies describing the usability evaluation of prototype app versions providing a personalized treatment schedule and an indoor navigation service for outpatients [87,88]. Moreover, both apps seem to be limited to this sole in-hospital purpose, without patient-centered information regarding their disease, and restricted to Android operating software systems. A study by Westphal et al [89] described a very promising web-based system for providing real-time information to ED patients regarding the procedures that they may encounter during their journey. However, similar to the previous 2 studies, this system focused only on the patient’s journey within the hospital and did not address the patient’s experience over the entire course of care.

The InfoKids app aims to bridge these gaps. Importantly, it is intended for wider use within our institution. Through the current iterative processes of development and evaluation, it is intended to soon become a more universal tool to connect the whole population seeking ED care (ie, adults, geriatric, and gynecologic) in a service area of more than 1 million people. In this sense, this study contributes to this iterative development process. Given its interconnection with the hospital’s computerized system, this app has the potential to ensure better coordination, continuity, and transition of care, thus improving both the patient experience and hospital efficiency.

### Strengths

This study had several strengths. To our knowledge, this was the first report of findings of the usability evaluation of an app supporting the longitudinal patient care transition from home up to ED discharge. Second, the mixed methods approach used in combination with different types of usability methods was another strength already identified by studies recognizing the utility of using qualitative and quantitative approaches for app usability testing [72,90]. Third, the 9 goal-oriented tasks assessed were centered around the main features of the app. The fact that users can perform a set of tasks centered around these features that are representative of those that users would normally use in clinical care was identified as a good way to determine the usability of the app and its features and workflow [91]. Fourth, this study added to the literature that recommends more usability studies focused on patient-centered apps [72,91-97]. It also contributed to the effort to publish usability studies based on academic development and patient-centered care, rather than a purely commercial development approach [42].

### Limitations

Our study had some limitations. First, we used an artificial laboratory environment, which has a low degree of fidelity. As a result, the generalizability and transferability of the results may be limited in real-life settings. Furthermore, the results obtained were based on an assessment of usability with participants who were naïve to its use. Therefore, it can be assumed that in-depth use of the app beforehand could have improved the perception of its usability among people who had used it before and avoided certain problems of comprehension and navigation. Interestingly, the use of a tutorial that was supposed to correct these problems seems to have been a source of difficulties for users in itself, if only to find it in the app. Therefore, it might be judicious in future versions to replace it with an interface offering contextual help on each page, rather than a long tutorial to memorize or search for. These assumptions should be addressed in future studies. Another limitation is that the small sample of 17 users might not have been sufficiently large to reveal all usability issues. However, it was assumed that 5 users are already a sufficient sample to reveal 85% of usability problems, whereas 15 users were sufficient to uncover almost 97% of problems [98]. In contrast, the fact that only one scenario was proposed to users in an arbitrary order set by the investigators raises concerns about the applicability of the results to any other clinical situations or navigational pattern in the app. This scenario was chosen to test most of the functions of the app according to a logical workflow model that parents wishing to consult with their child in the emergency room would follow. However, it cannot be excluded that other scenarios could have generated other navigation schemes and usability problems or facilitation. For example, if task 4a (evaluated as the most complicated task) had not been interposed between the choice of symptoms (task 3) and the announcement of departure to the ED (task 5) in this scenario, it is possible that no navigational problems would have occurred. It might be interesting in a future study to test the usability of the app based only on several standardized scenarios without predefined tasks. Instead, tasks and navigation would be left to users’ discretion, as in real life. Finally, as the InfoKids app is intended to be used in case of emergency (or at least perceived as such by parents), the quiet and nonstressful laboratory environment used in the study may appear to be a limitation. Guidelines for conducting usability testing recommend establishing a calm and relaxed atmosphere in which users can work without feeling stressed [99-101], although stress in usability testing has rarely been studied so far. One of the few existing studies by Janneck and Dogan [99] compared a usability test performed in a laboratory under calm and relaxed conditions with a test situation in which several stressors (time pressure, noise, and social pressure) were applied. They observed that participants under stressful conditions demonstrated poorer performance in the execution and accuracy of tasks and rated the usability and user experience of the software much more negatively. However, it should be noted that although various situations tend to elicit different patterns of stress responses, there are also individual differences in perceived and behavioral stress responses to the same situation [102]. Indeed, future research assessing the impact of stressors on the usability of InfoKids would provide valuable input for future development in the adult setting.

### Conclusions

The usability of mHealth apps is an important factor for their adoption and use. This study addresses a gap in the literature by reporting findings from a usability evaluation relevant to a patient-centered mobile app designed to support the entire emergency care process by assisting patients before, during, and after an ED visit. Our results show that the usability of the current version of InfoKids is rated as good to excellent by users. However, areas for app improvement are identified and mitigation measures are proposed. These usability problems will be addressed in updated releases of InfoKids and will be used to inform the development of its next version as a universal app for all patients seeking ED care. The next step would be to determine whether this mobile app benefits ED patient experience and ED efficiency in a real-life patient environment and clinical conditions. Given the paucity of research in this area, we conclude that our findings could also be useful in paving the way for further research on mobile apps aimed at supporting and accompanying patients in their care episodes.

## Appendix

Multimedia Appendix 1 Description of the expected handling tasks of the app and criteria for the determination of completion for each task.

Multimedia Appendix 2 Screenshot of the *family* page. On the *family* page, each child is represented by a card containing their photo, the child’s first name, and an *edit* button located at the bottom right of the card. To access the child’s profile and edit the data, it is necessary to click this button. As many children as necessary can be added.

Multimedia Appendix 3 Screenshot of the child’s profile page. The *chronic illnesses* button must be clicked to view the child’s chronic illnesses.

Multimedia Appendix 4 Screenshot of the *symptom* page.

Multimedia Appendix 5 Screenshot of the child selection page.

Multimedia Appendix 6 Screenshot of the *H* icon indicating the localization of the emergency department on the map.

Multimedia Appendix 7 The 10-item System Usability Scale questionnaire.

Multimedia Appendix 8 Time-based efficiency per task (N=17 participants).

Multimedia Appendix 9 Overall relative efficiency per task (N=17 participants).

Multimedia Appendix 10 Details regarding the usability problems identified.

## Abbreviations

ED: emergency department
ORE: overall relative efficiency
SUS: System Usability Scale
TBE: time-based efficiency
TCR: task completion rate
