# Supplementary material for: Usability Testing of a Patient-Centered Mobile Health App for Supporting and Guiding the Pediatric Emergency Department Patient Journey: Mixed Methods Study
Source: JMIR Pediatr Parent. 2022 Mar 15;5(1):e25540. doi: 10.2196/25540 (PMC8965675; doi:10.2196/25540)
Supplement: Multimedia Appendix 1 [file pediatrics_v5i1e25540_app1.docx]

| Tasks | Expected handling of the app | Completion criteria |
| --- | --- | --- |
| 1. Create a parental account | To use the app, participants must create an account by filling in the in-app parental information page dedicated to this purpose (eg, name, contact address, phone number, insurance number, family pediatrician, etc.). | Participants successfully filled in all fields (as the screen is displayed directly after the app installation, it could not be missed). |
| 2. Create a child profile | Participants must create and complete an 8-field profile for a child, by clicking on *family* and then *add a child* (Figures 3 & 4).  The *chronic illnesses* and *regular treatments* fields must be completed. | Participants successfully filled in all fields. |
| 3. Find *symptoms* page | To find information about the symptom “cough” by going first to the homepage and then browsing the symptom’s decision tree. | Symptom “cough” selected. |
| 4a. Find and understand *waiting times* page | To find the current occupancy of the ED waiting room by going to the *info* page and then the *waiting times* page. | *Waiting times* page reached and understood. |
| 4b. Find and understand *forecast* page | To find the ED attendance statistics over the last 5 days by going to the *info* page and then the *forecast* page. | *Forecast* page reached and understood. |
| 5. Inform of departure to ED | Participants must first click on the *I am going to the ED* button to indicate their choice to go to the ED because of their child’s worsening cough and health. Participants must then select which child in the family list is concerned. | Participants successfully clicked on the *I am going to the ED* button and selected the right child. |
| 6a. Find *tutorial* page | To find the *tutorial* page. | *Tutorial* page reached. |
| 6b. Find *map* page | To find the hospital location on the *map* page. | *Map* page reached and hospital localized. |
| 7. Find the *diagnostic* sheet | To find the diagnostic sheet by going first to the *info* page, then the *history* page, and finally select the diagnostic sheet. | Participants successfully selected the diagnostic sheet. |
