# Supplementary figures and images for: Usability Testing of a Patient-Centered Mobile Health App for Supporting and Guiding the Pediatric Emergency Department Patient Journey: Mixed Methods Study
Source: JMIR Pediatr Parent. 2022 Mar 15;5(1):e25540. doi: 10.2196/25540 (PMC8965675; doi:10.2196/25540)

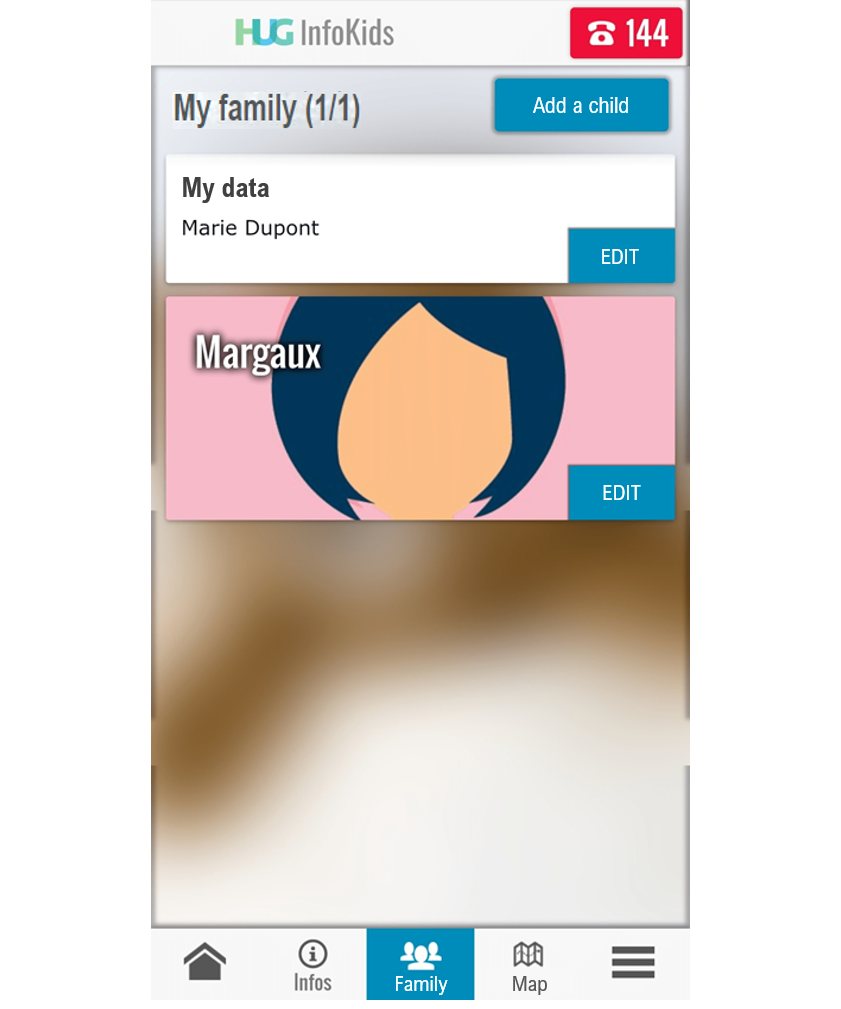

Supplement: Multimedia Appendix 2 [file pediatrics_v5i1e25540_app2.png]

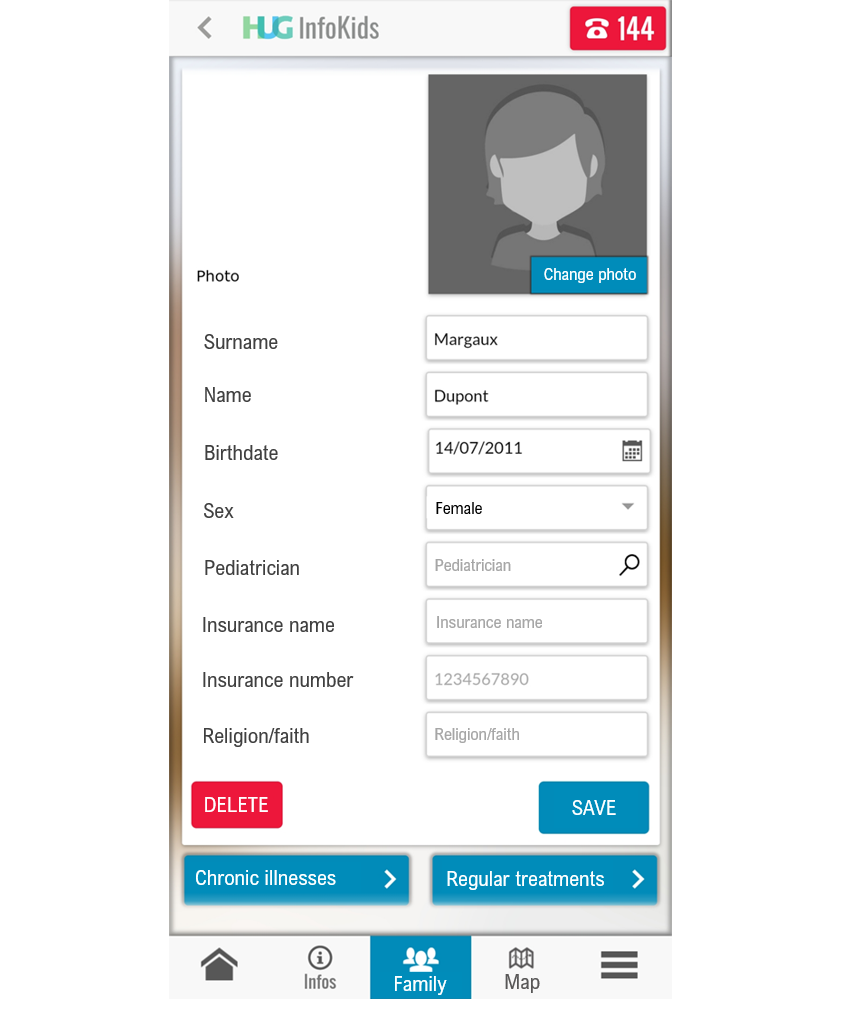

Supplement: Multimedia Appendix 3 [file pediatrics_v5i1e25540_app3.png]

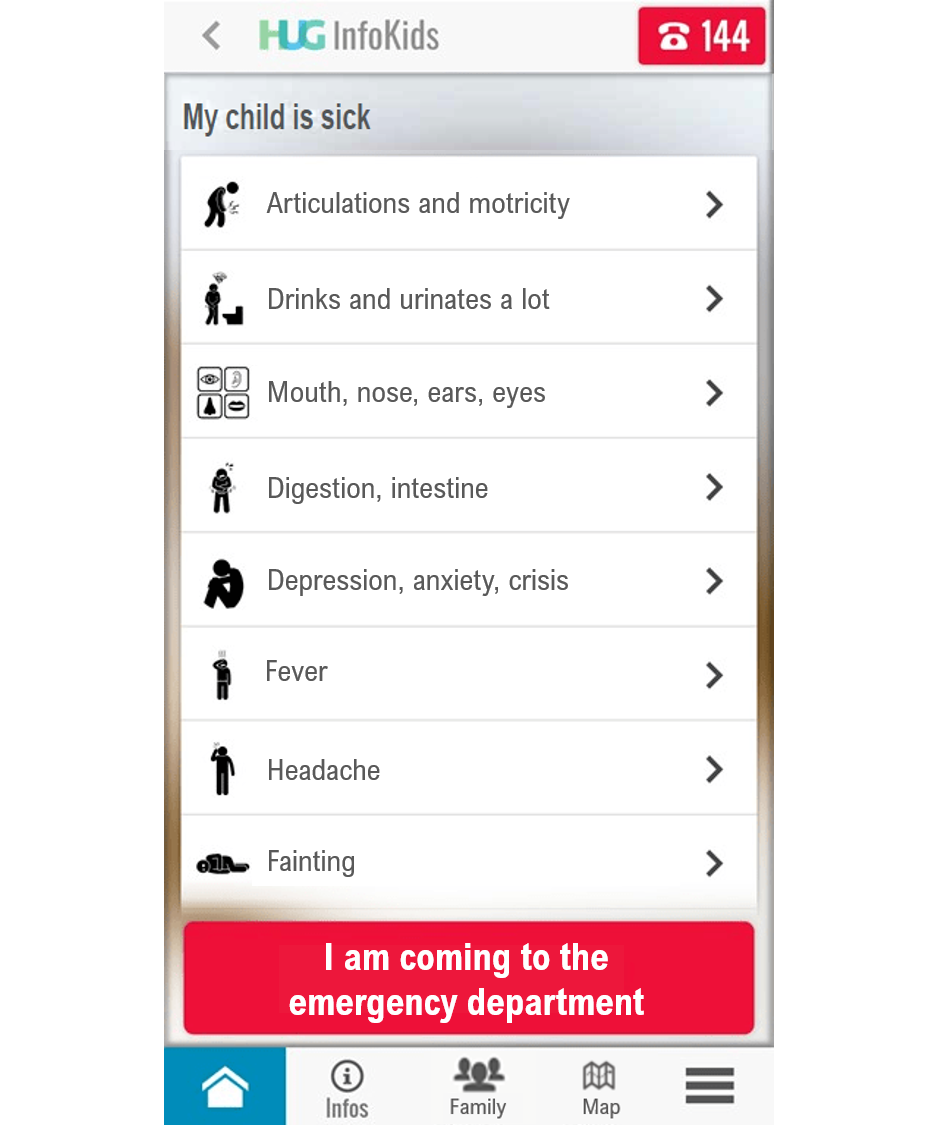

Supplement: Multimedia Appendix 4 [file pediatrics_v5i1e25540_app4.png]

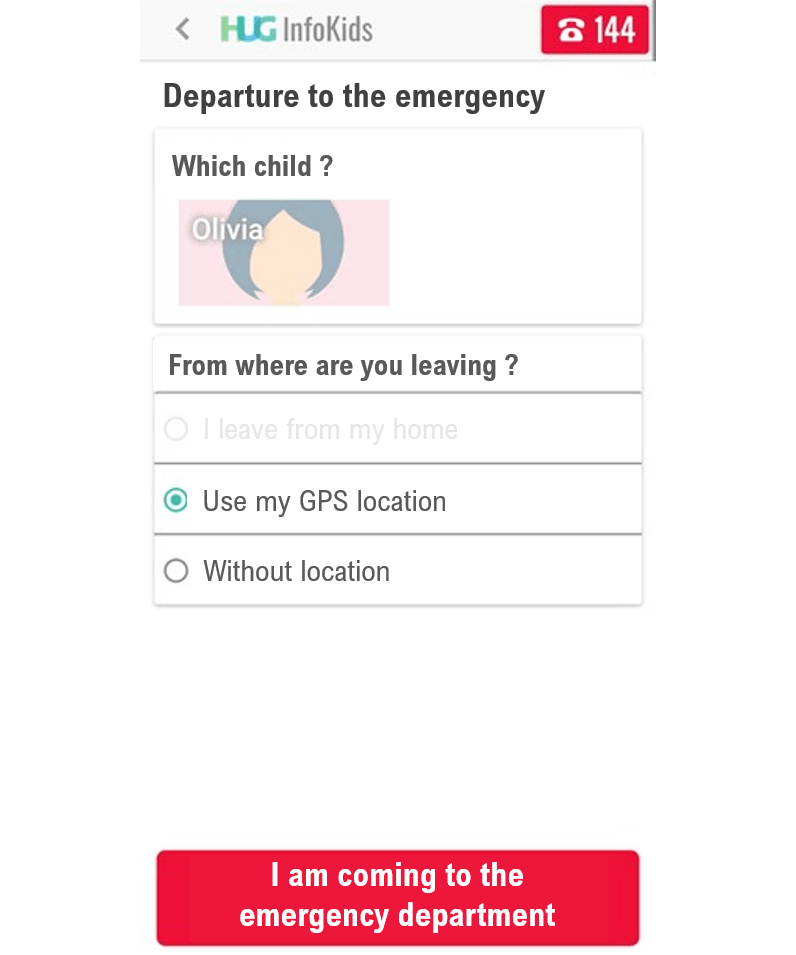

Supplement: Multimedia Appendix 5 [file pediatrics_v5i1e25540_app5.png]

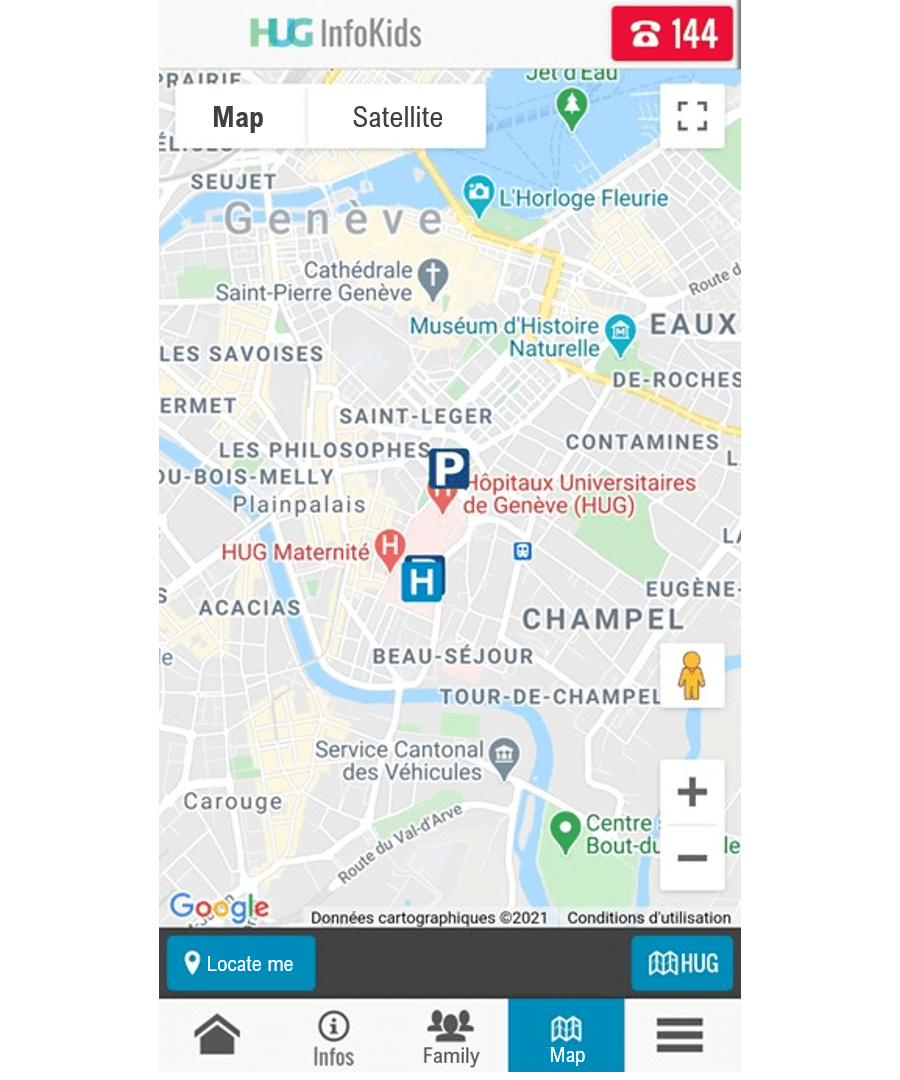

Supplement: Multimedia Appendix 6 [file pediatrics_v5i1e25540_app6.png]

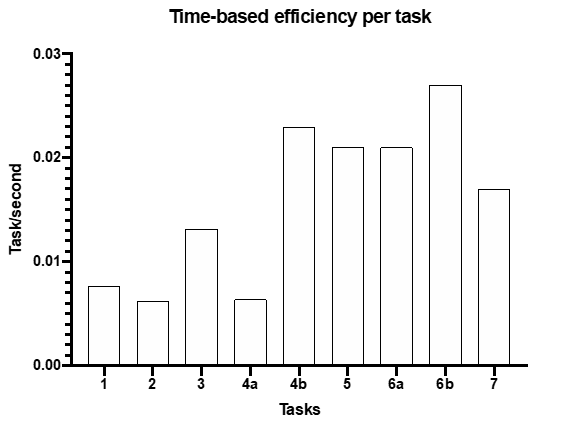

Supplement: Multimedia Appendix 8 [file pediatrics_v5i1e25540_app8.png]

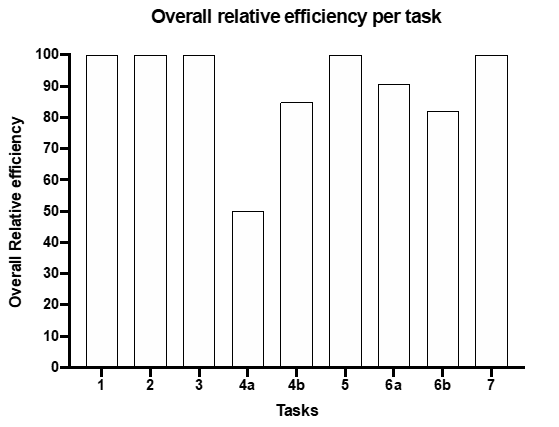

Supplement: Multimedia Appendix 9 [file pediatrics_v5i1e25540_app9.png]
