# Supplementary material for: Usability Testing of a Patient-Centered Mobile Health App for Supporting and Guiding the Pediatric Emergency Department Patient Journey: Mixed Methods Study
Source: JMIR Pediatr Parent. 2022 Mar 15;5(1):e25540. doi: 10.2196/25540 (PMC8965675; doi:10.2196/25540)
Supplement: Multimedia Appendix 7 [file pediatrics_v5i1e25540_app7.docx]

Q1: I think that I would like to use this system frequently

Q2: I found the system unnecessarily complex

Q3: I thought the system was easy to use

Q4: I think that I would need the support of a technical person to be able to use this system

Q5: I found that the various functions in this system were well integrated

Q6: I thought that there was too much inconsistency in this system

Q7: I would imagine that most people would learn to use this system very quickly

Q8: I found the system very cumbersome to use

Q9: I felt very confident using the system

Q10: I needed to learn a lot of things before I could get going with this system
