# Supplementary material for: Usability Testing of a Patient-Centered Mobile Health App for Supporting and Guiding the Pediatric Emergency Department Patient Journey: Mixed Methods Study
Source: JMIR Pediatr Parent. 2022 Mar 15;5(1):e25540. doi: 10.2196/25540 (PMC8965675; doi:10.2196/25540)
Supplement: Multimedia Appendix 10 [file pediatrics_v5i1e25540_app10.docx]

| Problems identified | | Frequency of users (%) | Severity score [62] | Ergonomic criteria [63] |
| --- | --- | --- | --- | --- |
| **Task 1. Parental account** | |  |  |  |
|  | None | 0 | 0 | - |
| **Task 2. Child profile** | |  |  |  |
|  | Access the child’s profile | 1 (6%) | 1 | Compatibility    The participant expected to access the child’s profile by clicking directly on the card (Multimedia Appendix 4). The procedure did not match user expectations. |
|  | Recording of information entered | 4 (24%) | 3 | Guidance – immediate feedback  Participants wondered if the information about the child’s chronic illnesses and regular medication had been properly saved (Multimedia Appendix 5). This information was not displayed directly on the child’s profile to inform the user that the entries were saved. |
| **Task 3. Information about a symptom** | |  |  |  |
|  | Find the *symptom* page | 5 (29%) | 2 | Significance of codes  Some participants did not directly find the menu tab that allowed to reach the symptoms’ list. Some participants were looking for this feature in the *infos* page, in the *tutorial*, or in the child's profile, instead of the *home* page (Multimedia Appendix 6). The icons and title of the menu tabs did not seem meaningful enough to allow an efficient navigation. |
|  | Find the *cough* symptom | 1 (6%) | 1 | Significance of codes  The participant did not find directly the *cough* symptom, searching first in the *mouth, nose, ears, eyes* category, before scrolling further to find the *cough, difficulty breathing* category (Multimedia Appendix 6). The title of the category *nose, mouth, throat* invited the user to search in this category. |
| **Task 4a. Waiting times** | |  |  |  |
|  | Find the page | 13 (76%) | 2.5 | Significance of codes  Some participants did not directly find the *waiting times* page, looking first into other pages, such as the *home* page, the *family* page, the hamburger menu, the *history* section in the *information* page, or by clicking on the button *I am coming to the ED* (Multimedia Appendix 7). The title of the menu tab *information* did not seem meaningful enough to allow an efficient navigation. |
|  | Understanding the screen displaying waiting times in the form of cars and colored lanes | 10 (59%) | 4 | Compatibility  Some participants faced difficulties to interpret and understand the meaning of the cars representing patients and the different colored lines representing the emergency levels (Multimedia Appendix 7). Participants had to interpret this information that was not presented directly in a usable form. |
| **Task 4b. Forecast** | |  |  |  |
|  | Find the page  The participants went first to the *history* page before finding the *forecast* page. | 4 (24%) | 4 | Significance of codes  Some participants had difficulties to find the *forecast* page, looking first at the *history* section of the *information* page (Multimedia Appendix 8). The title of the section *forecast* does not seem meaningful enough to allow an efficient navigation. |
| **Task 5. Indicate departure to the ED** | |  |  |  |
|  | Find the page | 7 (41%) | 3 | Compatibility  Some participants had difficulties to find the feature to inform about departure to the ED, looking first into the *information* or *map* tabs (Multimedia Appendix 9). They did not expect to find this feature in the *symptoms*’ list section. |
|  | Select the child | 13 (76%) | 2 | Guidance – prompting  As the child's card is already present in the form, it looks as if it is already selected and some participants did not understand that they had to select the concerned child (Multimedia Appendix 10). An error message asked users to select the child concerned if they did not do it, but users were not prompted to select the child directly. |
| **Task 6a. Map tutorial** | |  |  |  |
|  | Find the *map tutorial* | 6 (35%) | 4 | Compatibility  Some participants faced difficulties to find the *map tutorial*, looking first directly in the *map* page. The localization of the map tutorial in a separate page did not match user expectations (Multimedia Appendix 11). |
|  | Message hiding the button to access the *map tutorial* | 2 (12%) | 2 | Explicit control – user control  Some participants faced difficulties to find the map tutorial because of the pop-up message *You are expected at the ED* that hid the button to access the map tutorial (Multimedia Appendix 11). Some participants had difficulties to see the chevron that allows to reduce the message. The chevron does not seem visible enough to give control to the user. |
| **Task 6b. Location of the ED** | |  |  |  |
|  | Find the location of the ED | 9 (53%) | 4 | Significance of codes  Some participants did not understand the meaning of the ‘*H*’ icon indicating the localization of the ED on the map (Multimedia Appendix 12). This icon usually used to represent a hospital, but does not seem meaningful enough to indicate the ED. |
| **Task 7. Diagnostic sheets** | |  |  |  |
|  | Reach the *information* page | 4 (24%) | 2.5 | Compatibility  Some participants had difficulties to find the diagnostic sheet that was in the *history* section on the *information* page (Multimedia Appendix 13). They looked first in other sections such as the hamburger menu, the *home* tab or the *family* tab. The localization of the *diagnostic* sheet in the *information* page did not match user expectations. |
|  | Select the *history* section | 2 (12%) | 2 | Significance of codes  When reaching the *information* page, some participants had difficulties to find the correct section to access the *diagnostic* sheet. The title of the section *history* does not seem to be meaningful enough to allow an efficient navigation (Multimedia Appendix 13). |
